# Self-perceived oral health of people with and without diabetes mellitus: results of the study GEDA 2019/2020-EHIS

**DOI:** 10.25646/13080

**Published:** 2025-04-09

**Authors:** Laura Krause, Stefanie Seeling, Christin Heidemann

**Affiliations:** Robert Koch Institute, Department of Epidemiology and Health Monitoring, Berlin, Germany

**Keywords:** Diabetes mellitus, Oral health, Dental health, Self-assessment, Prevalence, Adults, Germany, Noncommunicable Diseases

## Abstract

**Background:**

Self-perceived oral health reflects the individual’s point of view. Both subjective (e.g. pain, aesthetic aspects) and objective criteria (e.g. oral diseases, functional limitations) are included in the assessment. Oral diseases interact with noncommunicable diseases such as diabetes mellitus.

**Method:**

Data basis is the study German Health Update (GEDA 2019/2020-EHIS). In the telephone interview, respondents were asked about the presence of diabetes in the last 12 months and the state of their teeth and gums. Prevalences, prevalence ratios (PR) and p-values from Poisson regressions were calculated for people aged 18 years and older (*N* = 22,613).

**Results:**

People with diabetes were more likely to rate their oral health as fair to very poor than people without diabetes (41.2 % vs. 27.5 %). The association between diabetes and fair to very poor self-perceived oral health persisted after statistical control for sociodemographic and behavioural characteristics (PR 1.22, *p* < 0.001). This applies to both women and men.

**Discussion:**

Based on a population-representative sample, it was shown for the first time for Germany that there is an association between diabetes and oral health in adulthood. These results support international research findings. Greater interdisciplinary cooperation between physician groups who treat people with diabetes and dentists is required.

## 1. Introduction

Oral health is an important component of general health, well-being and quality of life [1]. Self-perceived oral health reflects the individual view of people [2]. Both subjective criteria, e.g. pain and aesthetic aspects, and objective criteria, such as oral diseases and functional limitations, are included in the assessment [2]. Studies show that self-perceived oral health correlates with the objective oral health status [3–5]. In this respect, self-perceived oral health is an appropriate indicator for obtaining information on the oral health of the population as part of interview surveys [6].

The Robert Koch Institute (RKI) in Berlin (Germany) collects survey data on oral health and the utilisation of dental services as part of its health monitoring [6–9]. According to the study German Health Update (GEDA 2019/2020-EHIS), slightly more than a quarter of adults in Germany rate their oral health as fair to very poor, men more often than women [6]. In addition to male gender, older age and belonging to the low education group are also associated with fair to very poor self-perceived oral health. Other factors include behavioural aspects, e.g. daily smoking, and care-related aspects, e.g. non-annual utilisation of dental services and unmet dental care needs [6].

Furthermore, studies show that oral health is related to disease-related factors such as diabetes mellitus, cardiovascular and respiratory diseases [10]. Diabetes mellitus refers to a group of chronic metabolic diseases with an elevated blood glucose level as shared characteristic. The most common forms are type 1, type 2 and gestational diabetes [11]. Type 1 diabetes is an autoimmune disease that often develops in childhood and adolescence. Type 1 diabetes leads to a deficiency in the body’s own production of the hormone insulin, meaning that injections of insulin are vital to regulate glucose metabolism. Type 2 diabetes mainly manifests itself in middle and older age due to a reduced insulin sensitivity. In addition to a genetic predisposition, this is promoted by factors that can potentially be influenced, such as physical inactivity, unhealthy diet, overweight and smoking [11]. Type 2 diabetes therefore shares risk factors with oral health [10, 11]. Gestational diabetes first develops during pregnancy and usually disappears afterwards, but increases the risk of developing type 2 diabetes later on [11].

Study results point to a mutual relationship between oral health and diabetes: on the one hand, elevated blood glucose levels promote inflammatory processes in the gingiva (gums), on the other hand, bacteria from the periodontal pocket (gum pocket) can enter the bloodstream and negatively influence blood glucose control through inflammatory processes in the body [12, 13]. High blood glucose levels also damage the blood vessels, which increases the risk of secondary diseases of diabetes, e.g. cardiovascular complications, neuropathy or chronic kidney disease [14]. If blood glucose levels are adjusted through lifestyle changes or medication, this has a positive effect on oral health [15]. Conversely, an improvement in oral health is accompanied by an improved metabolic state [16, 17]. The S2k (i.e. consensus-based) guideline on *diabetes and periodontitis*, which was published in 2024 by the German Society of Dental and Oral Medicine, the German Society of Periodontology (DG PARO) and the German Diabetes Society (DDG), addresses the interrelationship between diabetes and oral health in detail [18].

Against this background, this article examines the self-perceived oral health of adults aged 18 years and older with and without diabetes in Germany.

## 2. Methods

### Study conduction and sample design

Data basis is the German Health Update (GEDA) – a nationwide, population-representative survey of the adult resident population living in Germany, which the RKI has conducted in several survey waves since 2008 [19]. The aim of the study is to provide current data on health status, health-related determinants and utilisation of the healthcare system for health reporting, health policy and public health research. Since the fourth wave, the questionnaire of the European Health Interview Survey (EHIS), which is mandatory for member states of the European Union, has been integrated into GEDA [20, 21]. This consists of four modules with questions on health status, health care, health determinants and social and demographic characteristics of the participants [22]. A detailed description of the EHIS study is published elsewhere [22]. Data basis for this analysis is the wave GEDA 2019/2020-EHIS, which took place as a telephone survey between April 2019 and September 2020. A total of 23,001 people aged 15 years and older were interviewed who could be reached either by landline or mobile phone (response rate: 21.6 %) [21]. As this article focuses on adulthood, data from 22,708 persons is available.

**Key messages**

► More than a third of women with diabetes (34.8 %) reported having fair to very poor oral health compared to around a fifth of women without diabetes (24.0 %).
► Among men, almost half of those with diabetes (46.5 %) reported fair to very poor oral health compared to around a third of those without diabetes (31.2 %).
► Women and men with diabetes were 1.18 times and 1.26 times more likely to have fair to very poor self-perceived oral health, respectively, after adjusting for sociodemographic and behavioural characteristics than those without diabetes.
► Greater interdisciplinary cooperation between physician groups treating diabetes and dentists is still necessary.

### 12-month prevalence of diabetes mellitus

After the introductory note: ‘This is about long-term diseases and chronic health problems. Please do not include temporary health problems.’, participants were asked: ‘Have you had any of the following diseases or complaints in the last 12 months?’. From a list of diseases, ‘diabetes, not gestational diabetes’ was one of the options. A dichotomous variable was created (yes/no) to distinguish respondents with diabetes from respondents without diabetes [23].

### Self-perceived oral health

After the introductory note: ‘Now comes a question about oral health.’, participants were asked: ‘How would you describe the state of your teeth and gums? Would you say it is ‘very good’, ‘good’, ‘fair’, ‘poor’, ‘very poor’. For the analyses, the response categories were combined into two categories: ‘very good or good’ and ‘fair to very poor’ [6].

### Statistical analysis

As a frequency measure, prevalences with 95% confidence intervals (95% CI) for fair to very poor self-perceived oral health were first calculated separately for persons with and without diabetes. To estimate the extent of the association between diabetes and self-perceived oral health, Poisson regressions were performed with oral health (fair to very poor vs. very good/good) as the dependent variable (outcome) and diabetes (yes vs. no) as the independent variable (exposure), which stepwise were adjusted for potentially influencing variables (confounders):

**Step 1**: unadjusted baseline models on the relationship between diabetes and self-perceived oral health
**Step 2**: statistical control for *gender* (collected as gender identity [24]), *age* (in categories recommended by the World Health Organization (WHO) for oral health: 18 – 34 years, 35 – 44 years, 45 – 64 years, 65 – 74 years, 75 years and older [25]) and *education* (in categories based on the ISCED classification: low, medium and high education group [26])
**Step 3**: additional statistical control for *current smoking* (yes/no) [27] and *daily consumption of sugar-sweetened beverages* (yes/no)
**Step 4**: additional statistical control for *Body Mass Index* (BMI, continuously included in the model) [28]

Prevalence ratios (PR) with 95 % confidence intervals and *p*-values are reported as the results of the regression models. A significant difference is assumed if the *p*-value is < 0.05. All analyses were performed with a weighting factor that corrects for deviations of the sample from the population structure (as of December 31, 2018) with regard to sex, age, district type, and education (ISCED). The district type reflects the degree of urbanisation and corresponds to the regional distribution in Germany. All analyses were carried out using the S TATA 17.0 survey procedures.

## 3. Results

The analyses are based on data from 22,613 people with valid information on the presence of diabetes in the last 12 months and on self-perceived oral health. Of the 22,613 people, 2,050 reported a diabetes. People with diabetes (65.7 years, 95 % CI: 64.6 – 66.8) were on average older than people without diabetes (50.2 years, 95 % CI: 49.8 – 50.6). The gender-specific analyses included 11,911 women and 10,642 men with valid information on gender identity.

Table 1 illustrates the relationship between diabetes and self-perceived oral health. People with diabetes rated their oral health more often as fair to very poor than people without diabetes (41.2 % vs. 27.5 %). The difference in self-perceived oral health between people with and without diabetes was evident in both genders, even though at a higher level in men: while 46.5 % of men with diabetes rated their oral health as fair to very poor, the figure for men without diabetes was 31.2 %. In contrast, 34.8 % of women with diabetes rated their oral health as fair to very poor compared to 24.0 % of women without diabetes. These differences are reflected in the unadjusted regression analyses (Model 1) and also remain after adjustment for sociodemographic characteristics (Model 2) and additional adjustment for behavioural characteristics (Model 3) as well as for BMI (Model 4). After adjustment for all characteristics (Model 4), the probability of a self-assessment of oral health as fair to very poor was 1.22 times higher (women: 1.18 times, men: 1.26 times) for people with diabetes (*p*-value < 0.05 in each case) than for those without diabetes (Table 1).

## 4. Discussion

Based on the nationwide, population-representative survey GEDA 2019/2020-EHIS, it was shown for the first time for Germany that significantly more adults with diabetes than without diabetes rate their oral health as fair to very poor. This difference was observed in both women and men. The association between self-reported diabetes and fair to very poor self-perceived oral health persisted even after controlling for sociodemographic and behavioural characteristics (gender, age, education, smoking, daily consumption of sugar-sweetened beverages, BMI).

The present results support international research findings that have investigated the relationship between diabetes and self-perceived oral health [29–32]: For example, a matched (i.e. paired) case-control study by age and gender from Portugal [31] investigated whether people with and without diagnosed type 2 diabetes differ in their self-perceived oral health. The results show that people with type 2 diabetes are less likely to rate their oral health as at least medium than people without type 2 diabetes. A study from Hungary [29] showed that people with self-reported diabetes were more likely to rate their oral health as bad than people without diabetes, even after controlling for sociodemographic and behavioural factors (gender, age, educational attainment, employment status, area of residence, smoking, alcohol consumption, BMI). A study from Denmark [30] investigated the relationship between self-reported type 2 diabetes and self-perceived oral health and found that people with type 2 diabetes were more likely to rate their oral health as poor than people without type 2 diabetes. This difference was also observed after adjustment for sociodemographic and behavioural characteristics (e.g. sex, age, educational attainment, smoking status, alcohol consumption, BMI). With regard to the cited studies, it should be noted that, in comparison to the present study, no results are shown separately for women and men. However, a study from France analyzed the relationship between diagnosed type 2 diabetes and self-perceived oral health in women and was able to show that women with type 2 diabetes report poor perceived oral health more frequently than women without type 2 diabetes, even after adjustment for various influencing factors (e.g. age, educational level, smoking status, physical activity, BMI, tooth brushing frequency, annual frequency of visit to the dentist, family history of diabetes) [32].

In addition, studies point to an association between diabetes and clinical parameters of oral health [10, 33, 34]. An association between type 2 diabetes and periodontitis is most frequently reported in the scientific literature [10]. There are also associations with other diseases of the oral cavity, such as caries, endodontic diseases (diseases of the tooth interior) and tooth loss [10, 33, 34]. In addition, there is an association between diabetes and changes to the oral mucosa, which in turn can lead to oral cavity carcinomas [15]. A systematic review provides evidence that there is already an association between diabetes and oral health in childhood and adolescence [35, 36].

In the present study, some strengths and limitations must be taken into account: The high number of participants is a strength of GEDA 2019/2020-EHIS. By applying a study-specific weighting factor, the results achieve a high degree of representativeness for the adult resident population living in Germany [21]. However, certain distortions, e.g. due to selective non-participation (selection bias), cannot be ruled out. As the study was conducted as a telephone survey, socially desirable response behaviour cannot be ruled out when answering the question on self-perceived oral health [37]. Furthermore, the question about the state of the teeth and gums only allows a general assessment of self-perceived oral health. A comprehensive and differentiated assessment is not possible because not all relevant aspects (e.g. the oral mucosa) are mentioned as examples or asked about individually. It should also be noted that gestational diabetes is already excluded by the question wording on diabetes in the present data set, but no distinction can be made between type 1 and type 2 diabetes. In addition, the telephone survey only allows the consideration of self-reported known diabetes. As there is often a latency period of several years between the manifestation and diagnosis of type 2 diabetes [38], it cannot be ruled out that people with unrecognised diabetes are included in the group of people without diabetes. However, such a misclassification tends to lead to an underestimation of the observed association. In the future, it will be possible to analyse the relationship between self-perceived oral health and medical diagnoses, such as diabetes, cardiovascular and respiratory diseases, on the data basis of the recently established RKI Panel Health in Germany [39].

## 5. Conclusion and outlook

Oral health is very important for personal well-being and quality of life [1]. In addition, there exist associations between oral health and diabetes and other noncommunicable diseases, such as cardiovascular and respiratory diseases [10]. Improvements in the quality of care in the prevention, early detection, diagnosis and treatment of diseases of the oral cavity therefore not only benefit oral health, but also the prevention and progression of diabetes by influencing the metabolic state [16]. In contrast, better blood glucose control in diabetes has a positive impact on oral health [15].

As it is possible to obtain indications of both the presence of diabetes and poor oral health through interviews, a promising approach lies in greater awareness of the mutual relationship between oral health and diabetes and greater interdisciplinary collaboration between GPs, diabetes specialists and dentists [40]. For example, questions about periodontal diseases (diseases of the periodontium) could be included in the routine examination of people with diabetes and, if symptoms are present, referred to the dental practice [40]. The Diabetes Mellitus Type 2 Disease Management Programme (DMP), a structured treatment programme for people with diabetes, recommends in this regard pointing out regular annual dental check-ups [41]. People without a diabetes diagnosis but with risk factors for type 2 diabetes should be advised by their dentists to undergo a medical check-up. Diabetes screening could also take place in the dental practice in order to refer patients to a diabetes care practice if the result is positive [40]. However, in Germany there is currently no legal basis for referring patients from a dental practice to a diabetes care practice and vice versa [40]. As an association between diabetes and oral health can be observed already in childhood and adolescence [35, 36], pediatricians should also be included in the considerations for strengthened interdisciplinary cooperation.

Qualitative studies from Germany show that there are deficits among respondents from the medical and dental professions with regard to the knowledge content of the other profession, e.g. on oral and systemic diseases, and that associations between these diseases are still too rarely communicated to patients [42, 43]. One reason for this is probably the separate medical education in Germany. In this respect, interprofessional education could help to improve cooperation in the future [42]. Overall, increased information is needed for the population and medical staff regarding existing associations between oral and systemic diseases [40]. The Federal Dental Association (BZÄK) in Germany aims to achieve this through the campaign https://paro-check.de, which also involves general practitioners and diabetologists [40]. Information and recommendations for medical staff can also be found in the new S2k guideline on diabetes and periodontitis mentioned above [18].

Finally, the results underline to focus on the prevention of classic risk factors for type 2 diabetes and oral health, such as physical inactivity, unhealthy diet, overweight and smoking [44]. In this regard, both behavioural and structural prevention measures play a role.

## Figures and Tables

**Table 1:** Association between diabetes and self-perceived oral health by gender in adults; prevalences in percent (%) and prevalence ratios (PR) with 95 % confidence intervals (95 % CI) and p-values from Poisson regressions. Source: GEDA 2019/2020-EHIS

Self-perceived oral health (fair to very poor)														
	Proportion		Model 1			Model 2			Model 3			Model 4		
	%	(95 % CI)	PR	(95 % CI)^1^	*p*-value^1^	PR	(95 % CI)^2^	*p*-value^2^	PR	(95 % CI)^3^	*p*-value^3^	PR	(95 % CI)^4^	*p*-value^4^
**Total (*n* = 22,613)**														
Without diabetes	27.5	(26.5 – 28.4)	Ref.		**< 0.001**	Ref.		**< 0.001**	Ref.		**< 0.001**	Ref.		**< 0.001**
With diabetes	41.2	(37.8 – 44.6)	1.50	(1.37 – 1.64)		1.25	(1.14 – 1.37 )		1.28	(1.17 – 1.41)		1.22	(1.11 – 1.35)	
**Women (*n* = 11,911**)														
Without diabetes	24.0	(22.7 – 25.2)	Ref.		**< 0.001**	Ref.		**0.011**	Ref.		**0.005**	Ref.		**0.035**
With diabetes	34.8	(30.2 – 39.7)	1.45	(1.26 – 1.68)		1.21	(1.05 – 1.41)		1.24	(1.07 – 1.44)		1.18	(1.01 – 1.38)	
**Men (*n* = 10,642)**														
Without diabetes	31.2	(29.7 – 32.7)	Ref.		**< 0.001**	Ref.		**< 0.001**	Ref.		**< 0.001**	Ref.		**< 0.001**
With diabetes	46.5	(41.8 – 51.2)	1.49	(1.33 – 1.67)		1.28	(1.14 – 1.44)		1.33	(1.18 – 1.49)		1.26	(1.12 – 1.42)	

^1^ unadjusted

^2^ adjusted for gender, age and education (total) or age and education (women, men)

^3^ additionally adjusted for smoking and daily consumption of sugar-sweetened beverages

^4^ additionally adjusted for BMI

*n* = number of cases, Ref. = reference
